# The Shenzhen neonatal ARDS cohort study: a multi-omics approach to elucidating regional epidemiology, refined phenotypes, and long-term outcomes

**DOI:** 10.3389/fped.2025.1684309

**Published:** 2025-11-10

**Authors:** Ruolin Zhang, Jie Shen, Linying Yang, Yanzhen Xu, Yanping Guo, Lichun Bai, Hanni Lin, Xianhong Chen, Yan Huang, Xin Guo, Zhangbin Yu, Jinxing Feng, Jun Chen

**Affiliations:** 1 Department of Neonatology, Shenzhen Nanshan Maternity and Child Healthcare Hospital, Shenzhen, Guangdong, China; 2 Department of Neonatology, Shenzhen Children’s Hospital, Shenzhen, Guangdong, China; 3 Department of Neonatology, Shenzhen People’s Hospital (The Second Clinical Medical College, Jinan University, The First Affiliated Hospital, Southern University of Science and Technology), Shenzhen, Guangdong, China; 4 Department of Neonatology, Peking University Shenzhen Hospital, Shenzhen, Guangdong, China; 5 Department of Neonatology, Shenzhen Guangming District People’s Hospital, Shenzhen, Guangdong, China; 6 Department of Neonatology, Shenzhen Luohu Hospital Group Luohu People’s Hospital, Shenzhen, Guangdong, China; 7 Department of Neonatology, Longgang District Central Hospital of Shenzhen, Shenzhen, Guangdong, China; 8 Department of Neonatology, Shenzhen City Baoan District Women’s and Children’s Hospital, Shenzhen, Guangdong, China; 9 Department of Neonatology, Longgang District Maternity & Child Healthcare Hospital of Shenzhen City, Shenzhen, Guangdong, China

**Keywords:** neonatal acute respiratory distress syndrome, montreux definition, prospective cohort, epidemiology, precision medicine, bioinformatics, machine learning, long-term outcomes

## Abstract

**Background:**

Neonatal Acute Respiratory Distress Syndrome (NARDS) is a critical contributor to neonatal morbidity and mortality, with a global health burden that varies significantly by region. The Montreux definition provides a unified diagnostic framework; however, a significant clinical paradox exists. A prospective cohort in China reported a NARDS mortality rate of 12.6%, which is notably lower than the 17%–24% reported in a large-scale international prospective study. The underlying reasons for this discrepancy remain to be elucidated, whether due to differences in etiology, clinical practice, or patient demographics.

**Methods:**

The Shenzhen Neonatal ARDS Cohort Study (SZ-NARDS) is a prospective, multicenter observational cohort study spanning from 2025–2028, designed to address this knowledge gap. We will enroll more than 1,000 neonates who meet the Montreux criteria across nine tertiary neonatal intensive care units (NICUs) in Shenzhen, China. Longitudinal data collection includes granular clinical parameters, respiratory support metrics, and multi-modal biospecimens for deep phenotyping and multi-omics profiling. Survivors will undergo rigorous follow-up until 36 months' corrected age, with standardized neurodevelopmental, pulmonary, and growth assessments.

**Results:**

The primary objective of this study is to characterize the epidemiology of NARDS in this regional population and to test the following hypotheses: (1) The true incidence, etiology, and mortality rates of NARDS in Shenzhen will differ from existing international and Chinese cohorts, and these differences can be systematically explained by specific clinical and demographic factors. A multi-modal predictive model that integrates early clinical variables with multi-omics biomarkers has the potential to accurately identify neonates at high risk for severe NARDS [oxygenation index (OI) ≥ 16] and long-term adverse outcomes [Area Under the Receiver Operating Characteristic Curve (AUROC) > 0.85].

**Conclusions:**

The SZ-NARDS cohort is uniquely positioned to resolve a major clinical contradiction in NARDS epidemiology. By integrating deep phenotyping with a longitudinal biobank and advanced machine learning algorithms, this initiative will generate a comprehensive dataset. This dataset will serve to refine existing prognostic models, identify regional disparities in disease biology, and inform the development of precision medicine interventions for this vulnerable population.

**Clinical Trial Registration:**

Chinese Clinical Trial Registry, identifier ChiCTR2400093854.

## 1 Introduction

Acute Respiratory Distress Syndrome (ARDS) was first described in 1967 as a severe respiratory condition characterized by reduced lung volumes, decreased lung compliance, and a significant ventilation-perfusion mismatch, ultimately leading to progressive hypoxemia and respiratory distress (1–3). ARDS affects individuals across all age groups, including adults (4), children (5), and neonates (6). However, the etiology, clinical presentation, treatment, and prognosis of neonatal ARDS (NARDS) differ significantly due to the unique developmental status of neonatal lungs and their immature immune function (7). Global data show that the mortality rate of NARDS is approximately 20% (8), and survivors often face long-term developmental impairments. This places a significant emotional and financial burden on families (9–12). These unique challenges highlight the critical importance of advancing research on NARDS.

The Montreux definition for NARDS, which was introduced in 2017, signified the initial phase of NARDS research, with a predominant focus on clinical and investigative purposes (6). Following the study protocol, the second phase of this project involves a prospective cohort study with two primary aims: first, to determine the prevalence of NARDS; and second, to characterize the epidemiology, clinical progression, treatment, and outcomes of neonates meeting the Montreux criteria. This methodological approach mirrors the processes followed after the establishment of the Berlin definition of ARDS for adults and the Pediatric Acute Lung Injury Consensus definitions for children (4, 13). However, it is noteworthy that no large-scale international NARDS registry has been established, with only two prospective cohort studies currently available. De Luca et al. (2022) (8) conducted the first international multicenter study, reporting a NARDS prevalence of 1.5% and a mortality rate of 17%–24%. They identified sepsis, aspiration syndromes, and pneumonia as the leading etiologies. Similarly, in 2023, Long Chen et al. (14) conducted a multicenter study in China, which reported a comparable prevalence (1.44%) but a strikingly lower mortality rate (12.6%), with pneumonia, asphyxia, and early-onset neonatal sepsis as the predominant causes. These findings underscore not only regional variations in NARDS prevalence, mortality, and etiological factors, but also reveal a critical clinical paradox: why does NARDS mortality in China appear significantly lower than the international average? Potential explanations for this discrepancy remain unexplored and may include: (1) differences in etiological distributions (e.g., a higher proportion of direct vs. indirect lung injury in Chinese cohorts); (2) variations in clinical management strategies (e.g., surfactant use, ventilation protocols); or (3) demographic factors (e.g., gestational age profiles, access to tertiary care). Resolving this contradiction is essential for refining our global understanding of NARDS pathophysiology and optimizing resource allocation. Nevertheless, a notable limitation in these studies was their emphasis on etiology and short-term outcomes, offering scant insight into the longer-term implications of the condition. To address these gaps, our study undertakes a 3-year, large-scale, multicenter cohort investigation. It will place particular emphasis on the long-term follow-up of patients at high risk of mortality and explicitly aim to dissect the drivers of the observed regional mortality disparity. This will offer a more comprehensive understanding of the natural history of NARDS, thereby expanding the current body of literature and providing mechanistic insights into how the Montreux definition applies across diverse healthcare settings.

Despite significant advancements in the definition and standardization of NARDS, critical challenges persist in accurately identifying severe cases and predicting clinical outcomes. The PaO_2_/FiO_2_ ratio, a cornerstone of the Berlin criteria for adult ARDS, has been shown to be suboptimal in the neonatal population due to its dependence on positive end-expiratory pressure (PEEP). Notably, studies have demonstrated that neonates requiring higher PEEP often display PaO_2_/FiO_2_ ratios comparable to those with milder lung injury, which can lead to diagnostic ambiguities and the potential for undertreatment in critical cases (15). The 2023 Pediatric Acute Lung Injury Consensus Conference (PALICC-2) criteria introduced severity-specific indices, such as PaO_2_/FiO_2_ or SpO_2_/FiO_2_ for mild cases and the oxygenation index (OI) or oxygen saturation index (OSI) for severe cases (16). However, neonatal-specific physiological factors, including the elevated oxygen affinity of fetal hemoglobin and circulatory variability in preterm infants, significantly undermine the reliability of these indices (17). The Montreux criteria represent an advancement by prioritizing the OI, which demonstrates a stronger correlation with morbidity and mortality (18). Nevertheless, their practical application in neonatal intensive care units (NICUs) is hindered by several constraints, including inconsistent access to arterial blood gas analysis and inaccuracies inherent in transcutaneous oxygen measurements in preterm neonates (19). Moreover, the Montreux criteria, predominantly derived from adult and pediatric ARDS data, lack a robust foundation of neonatal-specific evidence, thereby limiting their precision and generalizability. This issue is compounded by the substantial heterogeneity observed in NARDS, encompassing diverse etiologies, clinical trajectories, and treatment responses. Addressing these limitations necessitates large-scale, prospective studies aimed at refining diagnostic criteria and developing predictive models that are specifically tailored to the neonatal population.

The heterogeneity of NARDS presents another challenge in clinical practice, as no specific treatments have been approved, and current clinical trials have yet to generate sufficient evidence for evidence-based interventions. Biological samples, such as blood and tracheal aspirate, are critical for advancing our understanding of NARDS at the genetic and molecular levels. Embedding these samples into biobanks within registry systems can promote translational research, facilitating phenotypic analyses and personalized treatments for NARDS patients. Though translational research on ARDS in adults and children has made substantial progress (20–22), studies focused on NARDS remain limited (23–25). Expanding biobank initiatives globally, particularly in non-Western populations, is vital for capturing regional genetic and molecular variations that can inform clinical guidelines. Furthermore, modern computational tools like machine learning (ML) offer a powerful way to analyze biobank data, uncover novel patterns for risk stratification, and personalize treatment strategies (26, 27). In ARDS, ML-derived phenotypes and prediction rules hold promise for personalized care. Recent ARDS literature emphasizes that precision medicine approaches—guided by biomarkers and ML—can identify subgroups of patients likely to respond differently to therapies (28, 29). Leveraging insights into neonatal immune response and lung development, this study will utilize machine learning to construct prognostic models by integrating biomarkers with clinical variables unique to neonates, such as gestational age and perinatal risk factors. These underexplored parameters in existing ARDS prediction frameworks hold potential for enhancing diagnostic precision, optimizing clinical outcomes, and advancing personalized care for this highly vulnerable population.

The SZ-NARDS cohort study will formally be initiated in January 2025. This is the only large prospective, multicenter, open, longitudinal cohort study on NARDS in Shenzhen, China, a special economic zone city in China. The SZ-NARDS cohort study will recruit eligible individual NARDS patients and comprehensively collect their clinical data and specimens with longitudinal follow-up for future research. Herein, we describe the rationale and design of the SZ-NARDS registry.

## 2 Objective

### 2.1 Primary objective

The primary objective of the SZ-NARDS cohort study is to establish a prospective, multicenter registry to comprehensively characterize the epidemiology, clinical phenotypes, and short- and long-term outcomes of NARDS in a large, contemporary neonatal cohort within the Shenzhen region.

### 2.2 Secondary objectives

The secondary objectives are grouped into three key areas of investigation:

#### 2.2.1 Epidemiological and clinical characterization

1. To evaluate the prognostic performance and clinical utility of the Montreux NARDS definition within the Shenzhen cohort. This involves assessing its association with mortality, severe morbidity, and long-term neurodevelopmental impairment (NDI), thereby establishing a robust framework for subsequent deep phenotype analysis.
2. To determine the incidence, disease burden, and clinical characteristics of NARDS in participating NICUs, and to describe the short-term mortality and morbidity of affected infants.
3. To characterize the clinical trajectories of NARDS, including the time course from the inciting insult to key endpoints such as severe disease, the requirement for extracorporeal membrane oxygenation (ECMO), or death, and to analyze mortality patterns over a three-year follow-up period.

#### 2.2.2 Prognostic model development

To develop and validate robust prognostic models that can identify infants at high risk for adverse outcomes. This includes creating models for the early prediction of severe NARDS (sNARDS) and for long-term prognosis.

#### 2.2.3 Biomarker discovery and validation

To discover and validate novel biomarkers for NARDS by linking our longitudinal biobank to clinical outcomes. This includes:

1. Identifying predictive proteomic and metabolomic signatures for severe NARDS (sNARDS) using longitudinally collected plasma/serum samples.
2. Characterizing the local inflammatory milieu through transcriptomic analysis of tracheal aspirates.
3. Performing a genome-wide association study (GWAS) using genomic DNA from blood to identify genetic variants associated with NARDS susceptibility and severity.

## 3 Methods

### 3.1 Study design and setting

**Figure 1:**
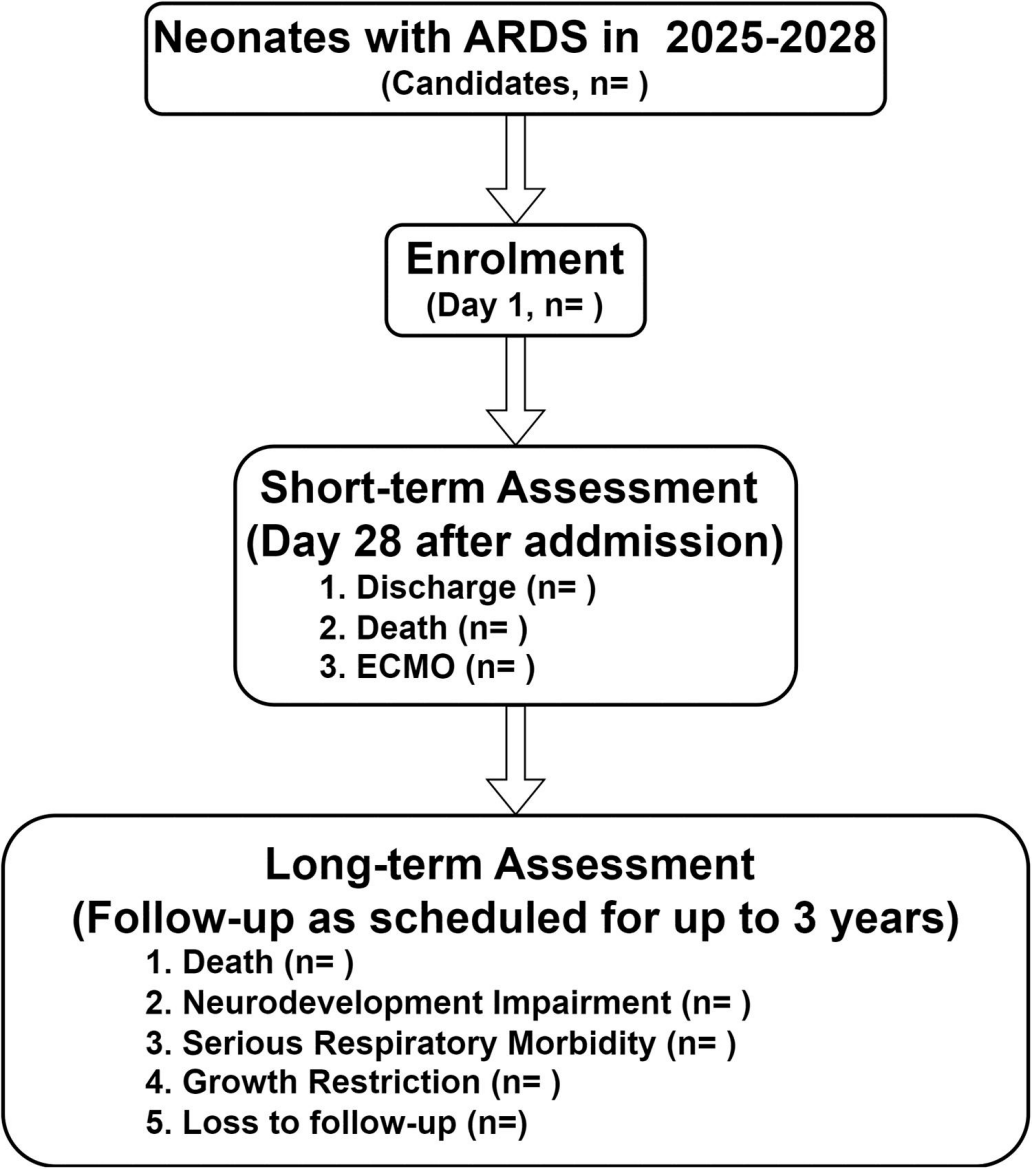
Flow chart of the study design, the SZ-NARDS, 2025-2028. Neonates diagnosed with ARDS were enrolled as participants, with clinical measurements and biological specimens collected on day 1, during the acute exacerbation phase (defined by the peak OI), on day 28, and within 24 h before death or initiation of ECMO. Outcome data, including censored cases, will be finalized in 2028 after completing follow-up through clinic visits or phone calls. ARDS, acute respiratory distress syndrome; ECMO, extracorporeal membrane oxygenation; SZ-NARDS, the Shenzhen neonatal acute respiratory distress cohort study; OI, oxygenation index.

The SZ-NARDS registry is a prospective, multicenter, observational cohort study, scheduled to run from January 1, 2025, to December 31, 2028. This study will be conducted across nine tertiary-level NICUs in Shenzhen, China, selected for their infrastructure, expertise in neonatal critical care, and patient volume to represent a broad spectrum of care in the region. The study protocol is reported in accordance with the Strengthening the Reporting of Observational Studies in Epidemiology (STROBE) guidelines (30) (Supplementary Material 1). An overview of the study design is provided in Figure 1.

Clinical management of patients will remain at the discretion of the local attending physicians according to institutional guidelines, with no study-specific interventions. The study features an open design, allowing for the potential inclusion of additional centers that meet the established criteria during the recruitment period.

### 3.2 Ethics and participant involvement

This study was approved by the Ethics Committee of Shenzhen Nanshan Maternal and Child Health Hospital and registered with the Chinese Clinical Trial Registry (ID: ChiCTR2400093854). Each participating center will obtain independent ethics committee approval before commencing recruitment. Participation will be entirely voluntary, and parents or legal guardians will receive comprehensive information on the study's objectives, procedures, and potential implications before providing written informed consent. No patients or members of the public were involved in the initial design or conception of this study.

### 3.3 Dissemination plan

The findings of this study will be disseminated through a multi-channel strategy aimed at both the public and the scientific community. Results will be shared with participating hospitals and the public via institutional health education platforms and social media. For the scientific community, outcomes will be presented at national academic conferences and submitted for peer-reviewed publication in reputable pediatric journals.

### 3.4 Confidentiality

To ensure the confidentiality of study participants, all documents related to the study will utilize a unique, site-specific participant identification number. All identifiable information will be stored separately from clinical data in a secure, encrypted, access-controlled database. The linkage file will be accessible exclusively to authorized site personnel.

### 3.5 Recruitment, enrollment, and termination

**Figure 2:**
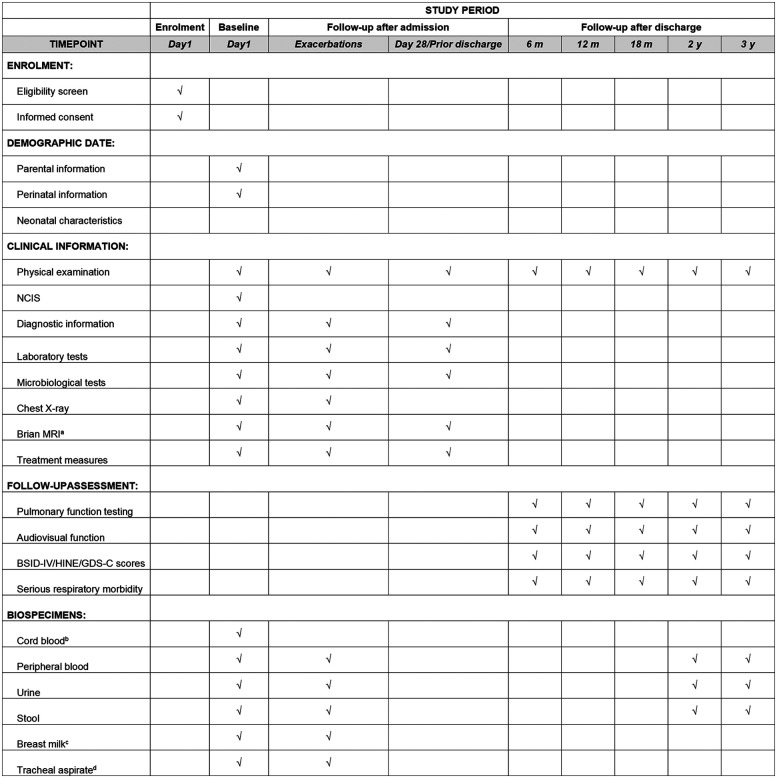
Enrolment campaign flow diagram, the SZ-NARDS, 2025-2028. Phase I: Recruitment of neonates diagnosed with ARDS; Phase II: Hospitalization follow-up, with data collection on day 1, during the acute exacerbation phase (defined by the peak OI), on day 28 (or the final hospital day if the stay was shorter), and within 24 h before death or initiation of extracorporeal membrane oxygenation (ECMO), if applicable; Phase III: Post-discharge follow-up conducted over three years according to Chinese guidelines for high-risk infants. ARDS, acute respiratory distress syndrome; ECMO, extracorporeal membrane oxygenation; SZ-NARDS, Shenzhen neonatal acute respiratory distress cohort study.

Recruitment for the SZ-NARDS cohort study will commence on January 1, 2025, at nine hospitals in Shenzhen, Guangdong Province, China. Enrollment and follow-up will be conducted concurrently, with recruitment continuing until the target sample size is achieved. The cohort will be considered complete once all participants have finished the 3-year follow-up period. Research coordinators will provide eligible parents with a detailed study pamphlet and will conduct face-to-face introductions to the cohort. A flowchart of the enrollment process is presented in Figure 2. A participant's active involvement in the study will cease upon death, guardian withdrawal of consent, or confirmed transfer to a non-participating medical facility where follow-up is not feasible.

### 3.6 Study participants and sample size

#### 3.6.1 Inclusion and exclusion criteria

Neonates will be enrolled consecutively upon meeting the full set of Montreux criteria for the diagnosis of NARDS during hospitalization. This cohort will include infants of all gestational ages, including premature infants, provided their respiratory distress meets all five Montreux criteria. These criteria include: (1) an acute onset of symptoms within one week following a known or suspected clinical insult; (2) exclusion of respiratory distress attributable to neonatal respiratory distress syndrome (NRDS), transient tachypnoea of the newborn (TTN), or congenital anomalies; (3) radiological evidence of bilateral, diffuse irregularities, including reduced translucency or opacities, that cannot be explained by conditions such as pleural effusion, atelectasis, NRDS, TTN, or congenital anomalies; (4) absence of congenital heart disease as the cause of pulmonary oedema, with echocardiographic confirmation required to verify the origin of the oedema; and (5) the presence of an oxygenation deficit, categorized by the OI as mild (4 ≤ OI < 8), moderate (8 ≤ OI < 16), or severe (OI ≥ 16). All five criteria must be fulfilled for diagnosis.

Exclusion criteria include: (1) respiratory distress due to primary surfactant deficiency (e.g., hyaline membrane disease), TTN, or congenital lung malformations; (2) congenital heart disease resulting in pulmonary oedema; (3) known genetic syndromes or chromosomal abnormalities; (4) data that did not conform to the Montreux criteria or cases with unavailable outcome data; and (5) abandonment or death occurring within 24 h of birth.

#### 3.6.2 Sample size determination and accrual plan

Although this is a descriptive cohort study, a formal sample size calculation was performed for a secondary objective of particular significance: the development of a multivariable prediction model for in-hospital mortality. In accordance with the widely accepted criterion of a minimum of 10 events per predictor variable for logistic regression, and anticipating up to 10 potential predictors, a minimum of 100 mortality events is required. Utilizing a conservative estimated mortality rate of 12.6% as derived from previous studies, it is estimated that a total cohort of at least 794 patients would be required to achieve the desired statistical power. To account for potential incomplete data and to ensure adequate power for other secondary analyses, the target sample size is set at 1,000 patients.

The enrollment target is supported by historical annual NICU admissions across the nine high-volume tertiary centers and by pilot accrual data from the coordinating center. Together, these data indicate feasibility within the planned four-year recruitment window (2025–2028). The enrollment-monitoring framework that will be implemented includes quarterly accrual reviews and formal feasibility assessments at 12 and 24 months. In the event that accrual falls below pre-defined thresholds, pre-specified contingency measures are to be enacted. The following measures are to be implemented: activation of up to 1–3 pre-identified tertiary centers in Shenzhen, Guangdong Province; temporary augmentation of research-coordinator full-time equivalents at underperforming sites to improve case ascertainment and data capture; and structured extension of the recruitment period, subject to steering-committee and ethics-committee approval. These measures are intended to ensure the viability of the study while maintaining methodological rigor.

### 3.7 Safety evaluation

As this is a non-interventional, observational study, participants will only receive standard clinical care. No study-specific procedures that pose additional risks are planned. Therefore, adverse events directly attributable to study participation are not anticipated. All clinical events and complications will be recorded as part of the routine data collection to characterize the natural history of the disease.

### 3.8 Data collection

**Figure 3:**
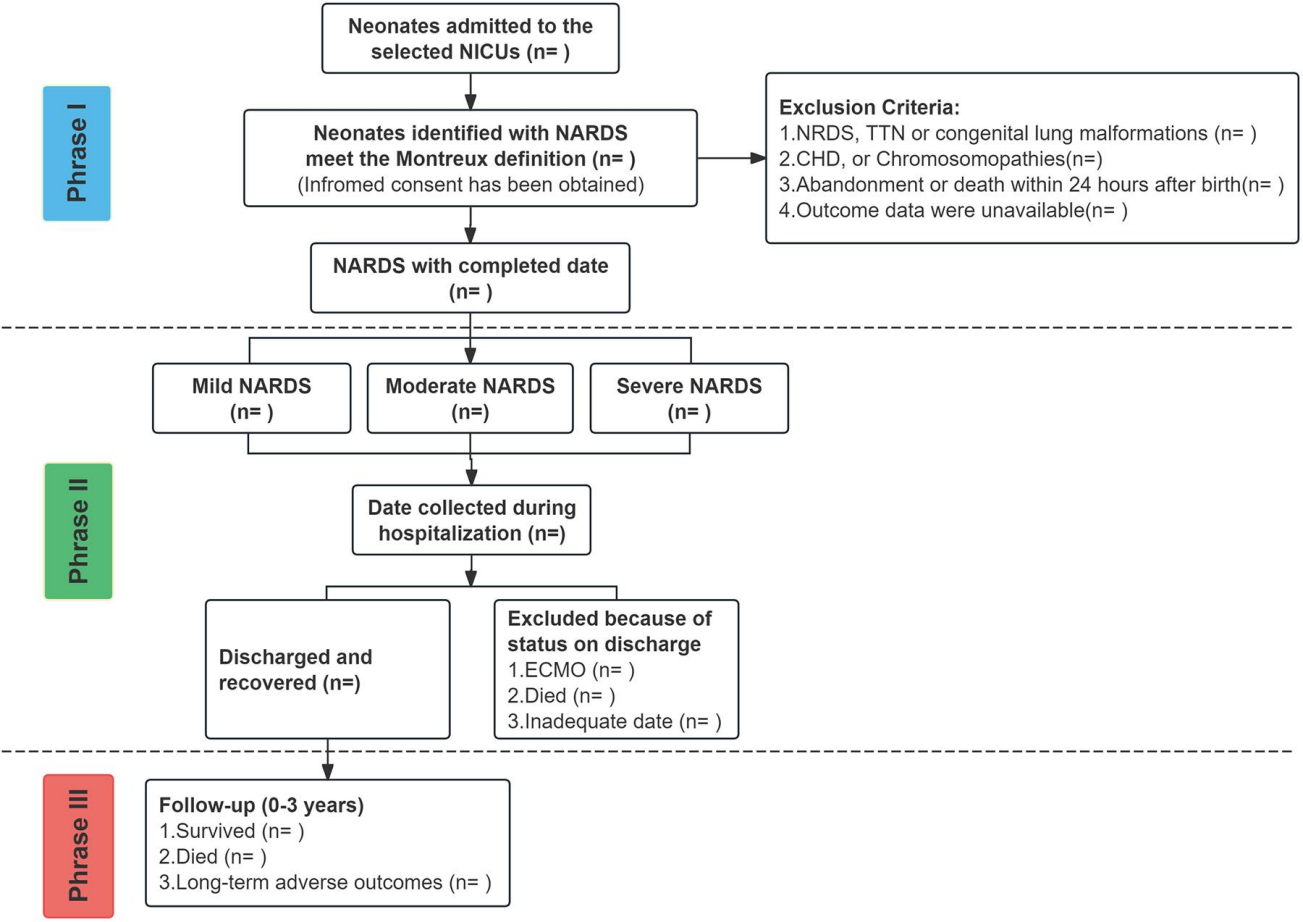
SZ-NARDS protocol time line spans from birth to three years of corrected age collecting health data and biospecimens. **(a)** MRI date will be collected if the infant is undergoing the procedure; **(b)** Cord blood, if available, will be bio banked at each participating center; **(c)** Breast milk samples will be collected if the infant is breastfed; **(d)** Tracheal aspirate samples will be collected when intubated infants require clinical suctioning. BSID-IV, the bayley scales of infant and toddler development, fourth edition; GDS-C, the griffiths developmental scales-Chinese; HINE, the hammersmith infant neurological examination; MRI, magnetic resonance imaging; NICS, neonatal intensive care severity score.

All data variables for the study are summarized in Table 1, with the study timeline illustrated in Figure 3.

**Table 1:** Details of measurements in the SZ-NARDS cohort study.

Phrase	Measurements
NICU Characteristics	Bed capacity, annual NICU admissions, availability of cardiac surgery, presence of in-house ICU physicians, ICU fellowship training programs, trauma care services, ECMO capabilities, high-frequency oscillatory ventilation (HFOV), respiratory therapists, burn care services, and point-of-care blood gas analysis.
Demographic data	
Guardian Information Perinatal information Neonatal characteristics	Age, gender, ethnicity, identification number, phone number, WeChat^a^ contact, home address, marital status, socioeconomic status, and educational attainment.
	Gravidity, parity, type of pregnancy, gestational weight gain, pre-existing maternal conditions, pregnancy-related complications, and treatments received.
	Gestational age, birth weight, gender, mode of delivery, multiple birth status, Apgar score, birth hospital, transport method, transfer duration, umbilical artery blood gas results, and admission temperature.
Baseline (day 1)	Physical examination Neonatal illness severity scores: SNAP-II, SNAPPE-II, NCIS Comorbidities: bacterial infection, systemic inflammatory response syndrome (SIRS), sepsis, severe sepsis, septic shock Laboratory tests: umbilical artery blood gas, complete blood count, CRP, PCT, standard blood gas analysis, lactate, liver and renal function tests, electrolytes, lipids, glucose, coagulation profile Microbiological cultures: blood, midstream urine (if urinary tract infection suspected), stool, sputum, and optional tracheal aspirate Imaging: chest x-ray, lung ultrasound, cranial ultrasound, echocardiography, and non-invasive cardiac function assessments Ventilation and treatment data: respiratory support parameters and pharmacologic interventions Biological specimens for DNA analysis: plasma (1–1.5 ml; from peripheral/cord blood), urine (1–1.5 ml), stool, sputum, breast milk, and optional tracheal aspirate (all stored at –80°C)
In-Hospital Follow-Up (At the most critical phase, Day 28, and within 24 h before death or ECMO initiation)	Identical to baseline measurements: physical examination, illness severity scores (SNAP-II, SNAPPE-II, NCIS), comorbidities, laboratory tests, microbiological cultures, imaging, ventilation/treatment data, and biological specimens.
Post-Discharge Follow-Up (Up to 36 months)	For deceased infants: date and primary cause of death For survivors: occurrence of major complications (e.g., severe neurodevelopmental impairment, chronic respiratory morbidity, growth restriction) For lost cases: date and reason for loss to follow-up

aEEG, amplitude integrated electroencephalogram; ARDS, acute respiratory distress syndrome; CRP, C-reactive protein; CT, computed tomography; ECMO, extracorporeal membrane oxygenation; HFOV, high-frequency oscillatory ventilation; MRI, magnetic resonance imaging; OI, oxygenation index; PBMC, peripheral blood mononuclear cells; PCT, procalcitonin.

a Wechat (Tencent Inc., Shenzhen, China) is a widely used social communication application in China, functionally similar to WhatsApp.

#### 3.8.1 Clinical data and key variable definitions

Demographic characteristics, baseline information, and follow-up data will be systematically recorded following admission and discharge. During hospitalization, data collected will include physical examination findings, neonatal critical illness scores, diagnoses, complications, biochemical parameters, pathogen cultures, imaging results, and treatments administered. Post-hospitalization follow-up assessments will be conducted at designated time points: on day 1 (within 24 h of NARDS diagnosis), during the acute exacerbation phase (defined as the moment when the OI reaches its peak), and on day 28 (or the final day of hospitalization if shorter than 28 days). For patients who succumb to their condition or require ECMO, data will also be gathered 24 h prior to death or the initiation of ECMO. Supplementary data, including hospitalization duration and associated costs, will also be documented.

Key clinical variables were standardized to ensure consistency in reporting:

1. Bronchopulmonary Dysplasia (BPD) will be diagnosed at 36 weeks postmenstrual age (PMA) and its severity classified according to the NICHD 2018 workshop definition, based on the level of respiratory support required (31).
2. Pulmonary air leak is defined as the presence of air in the lung parenchyma, detectable by radiographic imaging. This includes conditions such as pneumothorax, pneumomediastinum, interstitial emphysema, and pericardial emphysema.
3. Severe pulmonary hemorrhage is identified when hemorrhagic fluid is aspirated through endotracheal intubation, accompanied by acute respiratory distress and clinical deterioration. Radiographic findings typically reveal involvement of more than two lung lobes, and mechanical ventilation or other interventions are required within one hour of the appearance of blood.
4. Hemodynamically significant patent ductus arteriosus (hsPDA) is defined by echocardiographic confirmation of a ductus arteriosus with a diameter greater than 1.5 mm and a left-to-right transductal shunt (32).
5. Severe retinopathy of prematurity (ROP) includes stages 3, 4, or 5, or when surgical intervention is necessary (33).
6. Intraventricular hemorrhage (IVH) grade III affects more than 50% of the ventricular area and often results in lateral ventricle distention, while grade IV involves hemorrhagic infarction in the periventricular white matter.
7. Periventricular leukomalacia (PVL) is diagnosed based on cranial imaging findings, including ultrasound, computed tomography (CT), or magnetic resonance imaging (MRI).
8. Sepsis is classified as early-onset (within 72 h) or late-onset (after 72 h) based on the timing of bacteremia.

#### 3.8.2 Biospecimens collection and management

The collection of biospecimens is a key voluntary sub-study of the SZ-NARDS cohort. Participation requires a separate, specific written informed consent from the infant's legal guardians, ensuring they are fully aware of the distinct nature of the biobanking component.

##### 3.8.2.1 Ethics, consent, and incidental findings

A Standard Operating Procedure (SOP) for biospecimen handling has been approved by the lead hospital ethics committee and adopted by all participating centers (30). The biobank consent form describes the types of samples, processing methods, minimal risks, storage duration, and potential future unspecified research. A predefined policy for incidental findings (IFs), approved by the ethics committees, specifies which findings may be returned to families, the confirmation procedures in a clinical-grade laboratory, and the availability of genetic counseling. Guardians may opt in or out of receiving IFs and may withdraw consent at any time without affecting clinical care.

##### 3.8.2.2 Collection, processing, and storage

Where consented and feasible, six biospecimen types-cord blood, peripheral blood, urine, stool, breast milk, and tracheal aspirates-will be collected longitudinally at standardized phases of the disease trajectory, including baseline (within 24 h of NARDS diagnosis), acute exacerbation, day 28 or prior to discharge, and long-term follow-up visits (6–36 months). Each specimen is linked to specific downstream analyses: PAXgene Blood RNA tubes for transcriptomic profiling, buffy coat for genomic analysis, plasma/serum for proteomic and metabolomic assays, and stool/urine for microbiome and metabolic phenotyping. A detailed specimen collection schedule is presented in Figure 3. They will be processed within two hours, aliquoted, assigned anonymized IDs, and stored at −80 °C in a central biobank. Standardized SOPs will be used to minimize pre-analytic variability across all centers.

##### 3.8.2.3 Governance and future use

The SZ-NARDS biobank will be governed by a management committee. An independent scientific and ethics committee will also be established to review all proposals for future research using the stored samples. This structure ensures that samples are used only for scientifically and ethically sound research, such as genomic, proteomic, and biomarker analyses. All data generated from the biospecimens will be linked to the clinical data in the main registry through an anonymized code, thereby maintaining patient confidentiality.

#### 3.8.3 Post-discharge follow-up assessment

Survivors will be followed at corrected ages of 6, 12, 18, 24, and 36 months. Our primary long-term outcomes are survival status, neurodevelopmental impairment (NDI), severe respiratory morbidity (SRM), and growth restriction.

##### 3.8.3.1 Follow-up procedures and feasibility plan

To maximize the completeness of follow-up data, a multi-pronged strategy will be implemented. Each participating center will have a dedicated research nurse or coordinator responsible for patient tracking and communication. Families will be contacted via multiple channels (including WeChat, telephone, and SMS) for appointment reminders. To reduce the burden on families and improve adherence, research visits will be scheduled to coincide with routine high-risk infant follow-up clinics whenever possible, and a travel subsidy will be provided for study-specific visits.

##### 3.8.3.2 Outcome-specific assessments

1. Neurodevelopmental Impairment (NDI): NDI will be defined as the presence of one or more of the following at 36 months corrected age: (1) a score <85 on any of the cognitive, language, or motor composite scales of the Bayley Scales of Infant and Toddler Development, Fourth Edition (BSID-IV); (2) a diagnosis of cerebral palsy; or (3) severe hearing or vision impairment (bilateral deafness requiring aids or blindness) (34). To capture developmental trajectories, assessments will be performed longitudinally: the Hammersmith Infant Neurological Examination (HINE) will be used at 6 and 12 months, while both the Griffiths Developmental Scales-Chinese (GDS-C) and the BSID-IV will be administered at 24 and 36 months. Audiovisual evaluations will follow the Chinese High-Risk Infant Follow-Up Protocol, including auditory brainstem response (ABR) and visual evoked potential (VEP) testing at 6–12 months and behavioral audiometry and ophthalmologic examinations at 24 and 36 months.
2. Severe Respiratory Morbidity (SRM): SRM is defined by the need for tracheostomy, prolonged hospitalization for respiratory issues beyond 50 weeks postmenstrual age, or the requirement for supplemental oxygen, respiratory support, or respiratory monitoring, or multiple rehospitalizations for respiratory causes (35). Pulmonary function will be assessed using tidal respiratory analysis (36, 37), which evaluates parameters such as tidal volume, inspiratory/expiratory ratio, and peak tidal expiratory flow.
3. Growth Restriction: At each visit, weight, length, and head circumference will be measured and plotted on WHO growth charts for term infants and the Fenton growth charts for preterm infants. Growth restriction will be defined as weight or length below the 10th percentile for corrected age and sex.

#### 3.8.4 Data on confounding factors and NARDS etiology

Recognizing the significance of confounding factors, particularly in the premature infant population, the data will be stored in the “Perinatal Cloud Database” (https://www.perinatalcloud.com/). This system, developed by the study's principal investigator (ZY), utilizes an advanced online Case Report Form (CRF) designed to comprehensively capture potential confounders. Data collected will include detailed maternal history, gestational age, birth weight, and sex, along with significant neonatal comorbidities such as hsPDA, IVH, sepsis, and NEC. A comprehensive record of all treatment data will also be kept, including details regarding surfactant administration, ventilation strategies, and inotrope use. Further details about the database can be found in the Supplementary Material.

Furthermore, to address the underlying causes, each NARDS case will be classified as either “direct” (triggered by a primary pulmonary insult, such as pneumonia or meconium aspiration) or “indirect” (triggered by an extrapulmonary systemic insult, such as sepsis or perinatal asphyxia). This classification, which includes specific criteria for diagnoses like pneumonia (direct) or sepsis-associated NARDS (indirect), will be determined by the site investigator and reviewed for consistency by a central adjudication committee.

## 4 Outcomes

### 4.1 In-hospital outcomes

1. Primary in-hospital outcome: composite of mortality or requirement for ECMO within 28 days of NARDS diagnosis.
2. Secondary in-hospital outcomes include: 28-day mortality; 28-day ventilator-free days; duration of invasive mechanical ventilation; incidence of BPD, severe pulmonary hemorrhage, and air leak syndromes; and incidence of significant non-pulmonary organ dysfunction, including hsPDA, NEC (stage ≥ II), and severe IVH (grade ≥ III).

### 4.2 Post-discharge follow-up outcomes

1. Primary follow-up outcome: survival status at 36 months corrected age.
2. Secondary follow-up outcomes: NDI, SRM, growth restriction, and loss to follow-up rates (evaluated at the 24- and 36-month assessments).

### 4.3 Biomarker and predictive modeling outcomes

The primary outcomes related to the multi-omics biomarker analysis are:

1. The identification and validation of a proteomic and/or metabolomic signature, derived from baseline (within 24 h of diagnosis) plasma/serum samples, that demonstrates high accuracy [defined as an Area Under the Receiver Operating Characteristic Curve (AUROC) > 0.85] for predicting the development of severe NARDS (OI ≥ 16) within 72 h of diagnosis
2. The discovery of novel genetic variants *via* a Genome-Wide Association Study (GWAS) that are significantly associated with susceptibility to NARDS and/or in-hospital mortality.

## 5 Quality control

### 5.1 Data management system

This study will prospectively recruit patients, with recruitment anticipated to conclude in 2028. Data will be stored in the Perinatal Cloud Database, a platform accessible via both desktop and mobile devices. Each participating center and patient will be assigned a unique identification number. The online platform ensures secure data storage, facilitates data export based on specified search criteria, and incorporates a logical validation system that flags missing data, outliers, and inconsistencies. This system is designed to uphold data reliability, integrity, and accuracy, while supporting audit trails, data management, and source data validation. As participating centers utilize heterogeneous Electronic Health Record (EHR) systems, data are manually abstracted by trained research coordinators and entered into the central online Case Report Form (CRF). To ensure data fidelity, this process is supported by a rigorous, multi-tiered quality control and data validation protocol designed specifically to mitigate errors associated with manual entry.

### 5.2 Personnel training

Prior to the implementation of the electronic data capture system, comprehensive training will be conducted to ensure all personnel are adequately prepared. Upon completion of training, access rights to the corresponding system will be granted to relevant personnel.

### 5.3 Verification by regulatory authorities

A three-tiered quality control process has been established to ensure the accuracy of data entry at each hospital. Each hospital is required to designate a primary data quality controller to review all data entries. Additionally, the coordination center will appoint secondary data quality controllers at each hospital, who will sample and inspect a minimum of 30% of the data entries on a weekly basis. The Data Committee represents the third level of quality control, performing monthly reviews of at least 10% of the data entries.

To further enhance diagnostic consistency and minimize misclassification bias across centers, a central adjudication committee will be established. This committee, composed of at least two expert neonatologists blinded to the originating site, will independently review a random sample of 10% of all enrolled cases, as well as all ambiguous cases flagged by site investigators. This review ensures the uniform application of the Montreux criteria, particularly regarding radiographic interpretation and the exclusion of other etiologies. Furthermore, WeChat groups have been established at each hospital to provide real-time feedback on inspection results and facilitate necessary corrections.

### 5.4 Archiving of traceable raw data

Source-verified, anonymized copies of essential medical records will be securely archived digitally at the coordinating center. To ensure data security and redundancy, three separate backup copies will be maintained. Site-level investigators will have access only to their own center's data. The central data management committee will have monitored access to the complete dataset for quality control and unified statistical analysis.

## 6 Statistical analysis

### 6.1 Statistical analysis plan

Continuous variables will be summarized as mean ± standard deviation (SD) or median with interquartile range (IQR), depending on distribution. Categorical variables will be presented as frequencies and percentages. Group comparisons will be conducted using the student's *t*-test or Mann–Whitney *U* test for continuous variables, and the *χ*² test or Fisher's exact test for categorical variables.

Univariate logistic regression will be used to examine associations between NARDS and study outcomes. Multivariable logistic regression will then be performed to identify independent predictors. Missing data will be addressed primarily using multiple imputation by chained equations (MICE), assuming data are missing at random. Variables with excessive missingness will be excluded according to predefined thresholds, and the imputation model will be structured accordingly. Sensitivity analyses will include complete-case analysis, k-nearest neighbors (k-NN) imputation, and inverse probability weighting to evaluate the robustness of findings to loss to follow-up. A two-sided *p* < 0.05 will be considered statistically significant. All analyses and visualizations will be conducted using R software (version 4.3.3).

### 6.2 Machine learning and predictive modeling

Beyond conventional regression, predictive modeling will employ a suite of algorithms, including penalized logistic regression (Elastic Net), decision trees, random forests, gradient boosting machines, support vector machines, and artificial neural networks. Two primary prediction tasks will be addressed: Early identification of neonates at risk of severe NARDS (OI > 16 within 72 h of diagnosis); Long-term prognosis (mortality, NDI, SRM, or growth restriction at the 24- and 36-month corrected age). Candidate predictors will be selected based on clinical relevance and temporal availability (within 24 h for early warning models; within 7 days for prognostic models). Feature selection and regularization will primarily use Elastic Net. To identify novel NARDS endotypes, Integrative Multi-Omics Factor Analysis (MOFA) or similar unsupervised data integration approaches [e.g., sparse Partial Least Squares Discriminant Analysis (sPLS-DA)] will be employed to identify latent factors and co-varying molecular pathways across omics layers. These integrated molecular signatures will subsequently serve as input features for downstream machine learning models (e.g., Random Forest) to construct highly discriminative and biologically informed predictive classifiers.

### 6.3 Model evaluation and validation

Model performance will be evaluated using the area under the receiver operating characteristic curve (AUROC), sensitivity, specificity, calibration plots, Brier score, and, where applicable, precision–recall curves for imbalanced outcomes. Internal validation will be performed using a robust strategy: randomly partitioning the cohort into a 70% Discovery Set and a 30% Internal Validation Set at baseline. The validation set will serve as an independent internal check for model generalizability. We will also perform internal validation using cross-validation and bootstrap resampling, strictly adhering to the TRIPOD reporting guidelines for predictive models (38). Where feasible, temporal or center-level external validation (or internal holdout by center) will be undertaken to assess generalizability.

### 6.4 Multi-omics integration and validation

Biomarker discovery will follow a split-sample design, with the cohort randomly divided into a training set (70%) and an independent validation set (30%). Within each omics layer, initial feature selection will be conducted using multivariate analytical techniques such as PLS-DA. Subsequently, integrative multi-omics analyses will be performed using the DIABLO framework (Data Integration Analysis for Biomarker Discovery using a Longitudinal Approach) to construct predictive models that integrate molecular and clinical data. Model performance will be evaluated in the validation set using AUROC, sensitivity, specificity, and calibration metrics, ensuring rigorous validation of biomarker-based classifiers.

### 6.5 Control of confounding and subgroup analyses

Multivariable models will adjust for key covariates (gestational age in completed weeks, birth weight z-score, sex, major comorbidities). We will perform stratified analyses by gestational age (<32 weeks, 32–36 weeks, ≥37 weeks) and present both adjusted and stratified effect estimates. To address prematurity as a potential source of confounding and misclassification (NRDS vs. NARDS), the central adjudication committee will review all ambiguous preterm cases, evaluating the timing of respiratory failure onset, radiographic features, surfactant exposure/response, and clinical course. For non-randomized treatment comparisons (e.g., ventilation strategies, surfactant use), we will use propensity score methods (matching, inverse probability weighting) and, when appropriate, instrumental variable approaches to reduce treatment selection bias. Sensitivity analyses will include restriction to term infants and to preterm strata to evaluate effect modification by prematurity.

### 6.6 Biostatistical oversight

The statistical analysis plan has been reviewed and approved by an experienced biostatistician to ensure methodological rigor and adherence to best practices in predictive modeling and outcome assessment.

## 7 Discussion

The SZ-NARDS cohort study brings several novel strengths to the field of neonatal respiratory research. First, it is the first large-scale, multicenter prospective study of NARDS in Shenzhen based on the Montreux criteria. Previous key studies have been limited: the landmark international Pediatric Critical Care Medicine cohort included 239 infants (8), and the only published Chinese series of comparable size was retrospective (314 infants) (39). Unlike these earlier efforts, SZ-NARDS cohort systematically captures all gestational ages and ARDS subtypes across many regions, vastly increasing sample size and representativeness. We acknowledge the ambitious scale of the SZ-NARDS cohort. Its comprehensive design is made feasible by a major multi-year grant from the Shenzhen Nanshan District Major Science and Technology Project, supplemented by dedicated institutional resources at all participating centers. This financial structure fully supports all aspects of the study, from multi-center data collection and biobanking to the final three-year follow-up assessments. By prospectively enrolling infants using uniform Montreux-based definitions, our cohort will yield high-quality epidemiological data specific to Chinese neonates. Second, SZ-NARDS cohort integrates a formal biobank. We will store serial blood and airway specimens from enrolled patients, enabling molecular and genetic analyses of ARDS pathophysiology. This is unique among NARDS cohorts-for example, the recent transcriptomic study by Liu et al. (*N* = 48) highlighted age-dependent immune signatures in NARDS, but had no longitudinal bio samples (40). Our biorepository will support future biomarker discovery and mechanistic studies that are impossible in purely clinical databases. Third, the design includes long-term follow-up. While prior reports have focused on in-hospital outcomes, SZ-NARDS cohort will track surviving infants' respiratory health and neurodevelopment over time, clarifying the legacy of NARDS. Collectively, the multicenter design, standardized protocols, biobanking, and longitudinal follow-up establish SZ-NARDS cohort as a pioneering registry with substantial translational potential.

From a methodological standpoint, we have emphasized scientific rigor and reliability. The Montreux criteria specify exact radiographic, oxygenation, and timing requirements, which we enforce via training and central review to ensure consistent case ascertainment. Strict inclusion/exclusion criteria (e.g., excluding primary surfactant-deficiency RDS or transient tachypnea) maximize diagnostic specificity. Data are collected through a unified electronic system with built-in consistency checks, and periodic audits will monitor data quality. These measures minimize misclassification and bias. While treatment paradigms are not standardized, allowing for the capture of real-world practice variation for comparative effectiveness research, all procedures for diagnosis, data collection, and outcome assessment are rigorously harmonized across centers using detailed Standard Operating Procedures (SOPs). In summary, SZ-NARDS applies rigorous and harmonized procedures to ensure its findings are robust and reproducible.

SZ-NARDS will also supplement and extend previous findings. The international cohort by De Luca et al. reported a NARDS prevalence of 1.5% and survival rates of roughly 83% at 30 days (8). Our registry will test these figures in China: for example, early data from SZ-NARDS already suggest a somewhat higher incidence than the global average, possibly reflecting broader inclusion of perinatal cases. Similarly, Chinese prospective work found lower mortality (12.6%) (14) than the international study (17%–24%) (8), perhaps due to different case mix or care settings. By enrolling infants throughout Shenzhen, SZ-NARDS will help clarify the true NARDS mortality in China and explore reasons for any discrepancies. Compared with Chen et al.'s single-center retrospective, our prospective design will allow more accurate capture of risk factors (e.g., infection, birth complications) and outcomes. In addition, the Luo et al. study (*N* = 925, largely preterm) found that ARDS was mostly a late-preterm phenomenon and often managed with inhaled nitric oxide (41). SZ-NARDS will examine whether these patterns hold in a broader population including term infants.

While innovative, our study has limitations. A primary limitation is the potential for significant selection bias, as the study is confined to Shenzhen, a single, economically developed “special economic zone” in China. The healthcare resources, patient demographics, and socioeconomic status in this region are not representative of the broader diversity across China, including rural or less developed areas. This may result in a study cohort with better outcomes than the national average, and therefore, our findings must be interpreted with caution and require validation in more geographically and socioeconomically diverse populations before they can be generalized. Loss to follow-up is another concern in longitudinal work. We have instituted multiple strategies to minimize dropout and will address any residual missing data with modern statistical methods like multiple imputation and inverse probability weighting, accompanied by sensitivity analyses. While the multicenter nature of the study introduces heterogeneity in clinical management, this is also a methodological opportunity. By meticulously documenting variations in interventions (e.g., ventilation strategies, surfactant use), we can leverage advanced statistical methods, such as propensity score matching or inverse probability weighting, to conduct comparative effectiveness analyses in a real-world setting. This can generate valuable hypotheses for future randomized controlled trials.

## 8 Conclusion

In conclusion, the SZ-NARDS registry promises to fill critical knowledge gaps in NARDS. By generating high-quality clinical data and linked biospecimens, it will inform clinical practice, guide the design of interventional trials, and enable biomarker discovery. From a translational perspective, the stored samples will support research into inflammatory mediators and genetic predispositions. Ultimately, by identifying distinct clinical and biological phenotypes of NARDS, this research aims to identify patient subgroups that may respond differently to specific therapies, paving the way for targeted interventions and the design of precision medicine trials for this vulnerable population. Overall, SZ-NARDS aims to translate the burden of NARDS into actionable knowledge: closing the loop from epidemiology to bench to bedside and back, thus advancing care for this vulnerable population.

## References

[1] Ashbaugh DG Bigelow DB Petty TL Levine BE. Acute respiratory distress in adults. Lancet. (1967) 2:319–23. 10.1016/s0140-6736(67)90168-7 4143721

[2] McNicholas BA Rooney GM Laffey JG. Lessons to learn from epidemiologic studies in ards. Curr Opin Crit Care. (2018) 24:41–8. 10.1097/MCC.0000000000000473 29135617

[3] Meyer NJ Gattinoni L Calfee CS. Acute respiratory distress syndrome. Lancet. (2021) 398:622–37. 10.1016/S0140-6736(21)00439-6 34217425 PMC8248927

[4] Force ADT Ranieri VM Rubenfeld GD Thompson BT Ferguson ND Caldwell E Acute respiratory distress syndrome. Jama. (2012) 307:2526–33. 10.1001/jama.2012.5669 22797452

[5] Khemani RG Smith LS Zimmerman JJ Erickson S. Pediatric acute respiratory distress syndrome. Pediatr Crit Care Med. (2015) 16:S23–40. 10.1097/PCC.0000000000000432 26035358

[6] De Luca D van Kaam AH Tingay DG Courtney SE Danhaive O Carnielli VP The montreux definition of neonatal ards: biological and clinical background behind the description of a new entity. Lancet Respir Med. (2017) 5:657–66. 10.1016/S2213-2600(17)30214-X 28687343

[7] Davis RP Mychaliska GB. Neonatal pulmonary physiology. Semin Pediatr Surg. (2013) 22:179–84. 10.1053/j.sempedsurg.2013.10.005 24331091

[8] De Luca D Tingay DG van Kaam AH Courtney SE Kneyber MCJ Tissieres P Epidemiology of neonatal acute respiratory distress syndrome: prospective, multicenter, international cohort study. Pediatr Crit Care Med. (2022) 23:524–34. 10.1097/PCC.0000000000002961 35543390

[9] Boucher PE Taplin J Clement F. The cost of ards: a systematic review. Chest. (2022) 161:684–96. 10.1016/j.chest.2021.08.057 34478719

[10] Gorman EA O Kane CM McAuley DF. Acute respiratory distress syndrome in adults: diagnosis, outcomes, long-term sequelae, and management. Lancet. (2022) 400:1157–70. 10.1016/S0140-6736(22)01439-8 36070788

[11] Lee SW Loh SW Ong C Lee JH. Pertinent clinical outcomes in pediatric survivors of pediatric acute respiratory distress syndrome (pards): a narrative review. Ann Transl Med. (2019) 7:513. 10.21037/atm.2019.09.32 31728366 PMC6828794

[12] Watson RS Beers SR Asaro LA Burns C Koh MJ Perry MA Association of acute respiratory failure in early childhood with long-term neurocognitive outcomes. Jama. (2022) 327:836. 10.1001/jama.2022.1480 35230393 PMC8889465

[13] Khemani RG Smith L Lopez-Fernandez YM Kwok J Morzov R Klein MJ Paediatric acute respiratory distress syndrome incidence and epidemiology (pardie): an international, observational study. Lancet Respir Med. (2019) 7:115–28. 10.1016/S2213-2600(18)30344-8 30361119 PMC7045907

[14] Chen L Li J Shi Y. Clinical characteristics and outcomes in neonates with perinatal acute respiratory distress syndrome in China: a national, multicentre, cross-sectional study. Eclinicalmedicine. (2023) 55:101739. 10.1016/j.eclinm.2022.101739 36386029 PMC9661498

[15] Cabello B Thille AW. Are we able to optimize the definition and diagnosis of severe acute respiratory distress syndrome? Med Intensiva. (2012) 36:322–3. 10.1016/j.medin.2012.02.006 22425339

[16] Emeriaud G López-Fernández YM Iyer NP Bembea MM Agulnik A Barbaro RP Executive summary of the second international guidelines for the diagnosis and management of pediatric acute respiratory distress syndrome (palicc-2). Pediatr Crit Care Med. (2023) 24:143–68. 10.1097/PCC.0000000000003147 36661420 PMC9848214

[17] Saugstad OD Aune D. Optimal oxygenation of extremely low birth weight infants: a meta-analysis and systematic review of the oxygen saturation target studies. Neonatology. (2014) 105:55–63. 10.1159/000356561 24247112

[18] Liu L Wang Y Zhang Y He Y Chen L Li F Comparison of the montreux definition with the Berlin definition for neonatal acute respiratory distress syndrome. Eur J Pediatr. (2023) 182:1673–84. 10.1007/s00431-023-04848-x 36735062

[19] Seethala RR Hou PC Aisiku IP Frendl G Park PK Mikkelsen ME Early risk factors and the role of fluid administration in developing acute respiratory distress syndrome in septic patients. Ann Intensive Care. (2017) 7:11. 10.1186/s13613-017-0233-1 28116595 PMC5256622

[20] Lin S Yue X Wu H Han T Zhu J Wang C Explore potential plasma biomarkers of acute respiratory distress syndrome (ards) using gc–ms metabolomics analysis. Clin Biochem. (2019) 66:49–56. 10.1016/j.clinbiochem.2019.02.009 30779905

[21] Viswan A Ghosh P Gupta D Azim A Sinha N. Distinct metabolic endotype mirroring acute respiratory distress syndrome (ards) subphenotype and its heterogeneous biology. Sci Rep. (2019) 9:2108. 10.1038/s41598-019-39017-4 30765824 PMC6375936

[22] Xu J Pan T Qi X Tan R Wang X Liu Z Increased mortality of acute respiratory distress syndrome was associated with high levels of plasma phenylalanine. Respir Res. (2020) 21:99. 10.1186/s12931-020-01364-6 32354336 PMC7193408

[23] Liu H Li J Guo J Shi Y Wang L. A prediction nomogram for neonatal acute respiratory distress syndrome in late-preterm infants and full-term infants: a retrospective study. Eclinicalmedicine. (2022) 50:101523. 10.1016/j.eclinm.2022.101523 35784441 PMC9241127

[24] Shen L Cai N Wan S Chen S. Development and validation of a predictive model for early diagnosis of neonatal acute respiratory distress syndrome based on the montreux definition. Front Pediatr. (2023) 11:1276915. 10.3389/fped.2023.1276915 38027256 PMC10652555

[25] Wu H Hong X Qu Y Liu Z Zhao Z Liu C The value of oxygen index and base excess in predicting the outcome of neonatal acute respiratory distress syndrome. J Pediatr (Rio J). (2021) 97:409–13. 10.1016/j.jped.2020.07.005 32822669 PMC8166491

[26] Greener JG Kandathil SM Moffat L Jones DT. A guide to machine learning for biologists. Nat Rev Mol Cell Biol. (2022) 23:40–55. 10.1038/s41580-021-00407-0 34518686

[27] Vamathevan J Clark D Czodrowski P Dunham I Ferran E Lee G Applications of machine learning in drug discovery and development. Nat Rev Drug Discov. (2019) 18:463–77. 10.1038/s41573-019-0024-5 30976107 PMC6552674

[28] Al-Husinat L Azzam S Al SS Araydah M Battaglini D Abushehab S A narrative review on the future of ards: evolving definitions, pathophysiology, and tailored management. Crit Care. (2025) 29:88. 10.1186/s13054-025-05291-0 39994815 PMC11852867

[29] Talebi H Dastgheib SA Vafapour M Bahrami R Golshan-Tafti M Danaei M Advancements in biomarkers and machine learning for predicting of bronchopulmonary dysplasia and neonatal respiratory distress syndrome in preterm infants. Front Pediatr. (2025) 13:1521668. 10.3389/fped.2025.1521668 40352605 PMC12062013

[30] He S Peng H Zhou P Hu F Yan X Su Q Multicentre online registration of bronchopulmonary dysplasia in very preterm infants in China: protocol for a prospective, open, observational cohort study. Bmj Open. (2024) 14:e85560. 10.1136/bmjopen-2024-085560 PMC11660318 39581740

[31] Higgins RD Jobe AH Koso-Thomas M Bancalari E Viscardi RM Hartert TV Bronchopulmonary dysplasia: executive summary of a workshop. J Pediatr. (2018) 197:300–8. 10.1016/j.jpeds.2018.01.043 29551318 PMC5970962

[32] van den Anker J. Expectant management or early ibuprofen for patent ductus arteriosus. N Engl J Med. (2023) 388:1917–8. 10.1056/NEJMc2304388 37195958

[33] Chiang MF Quinn GE Fielder AR Ostmo SR Paul CR Berrocal A International classification of retinopathy of prematurity, third edition. Ophthalmology. (2021) 128:e51–68. 10.1016/j.ophtha.2021.05.031 34247850 PMC10979521

[34] Romeo DM Ricci M Mirra F Venezia I Mallardi M Pede E Longitudinal cognitive assessment in low-risk very preterm infants. Medicina (B Aires). (2022) 58:133. 10.3390/medicina58010133 PMC8778540 35056441

[35] Jensen EA Dysart K Gantz MG McDonald S Bamat NA Keszler M The diagnosis of bronchopulmonary dysplasia in very preterm infants. An evidence-based approach. Am J Respir Crit Care Med. (2019) 200:751–9. 10.1164/rccm.201812-2348OC 30995069 PMC6775872

[36] Jat KR Agarwal S. Lung function tests in infants and children. Indian J Pediatr. (2023) 90:790–7. 10.1007/s12098-023-04588-8 37261706 PMC10233185

[37] Sly PD Tepper R Henschen M Gappa M Stocks J. Tidal forced expirations. Ers/ats task force on standards for infant respiratory function testing. European respiratory society/American thoracic society. Eur Respir J. (2000) 16:741–8. 10.1034/j.1399-3003.2000.16d29.x 11106222

[38] Collins GS Moons K Dhiman P Riley RD Beam AL Van Calster B Tripod + ai statement: updated guidance for reporting clinical prediction models that use regression or machine learning methods. Br Med J. (2024) 385:e78378. 10.1136/bmj-2023-078378 PMC11019967 38626948

[39] Guo JY Chen L Shi Y. A single-center retrospective study of neonatal acute respiratory distress syndrome based on the montreux definition. Zhongguo Dang Dai Er Ke Za Zhi. (2020) 22:1267–72. 10.7499/j.issn.1008-8830.2007027 33327996 PMC7735934

[40] Liu C Ai Q Yang J Fan Y Jia W Si L Transcriptomic signatures of neonatal acute respiratory distress syndrome in a prospective cohort of respiratory distress. Iscience. (2025) 28:113007. 10.1016/j.isci.2025.113007 40697828 PMC12281067

[41] Luo J Chen J Li Q Feng Z. Differences in clinical characteristics and therapy of neonatal acute respiratory distress syndrome (ards) and respiratory distress syndrome (rds): a retrospective analysis of 925 cases. Med Sci Monit. (2019) 25:4992–8. 10.12659/MSM.915213 31278248 PMC6636403

