# Supplementary material for: The Shenzhen neonatal ARDS cohort study: a multi-omics approach to elucidating regional epidemiology, refined phenotypes, and long-term outcomes
Source: Front Pediatr. 2025 Nov 10;13:1684309. doi: 10.3389/fped.2025.1684309 (PMC12640991; doi:10.3389/fped.2025.1684309)

Clinical Data Management

The data will be stored in a perinatal cloud database developed by the sponsor (ZY) (https://www.perinatalcloud.com/). Perinatal Cloud Database Filling Steps as follow:

STEP 1 Enter the hospital's dedicated account and password to log into the database.

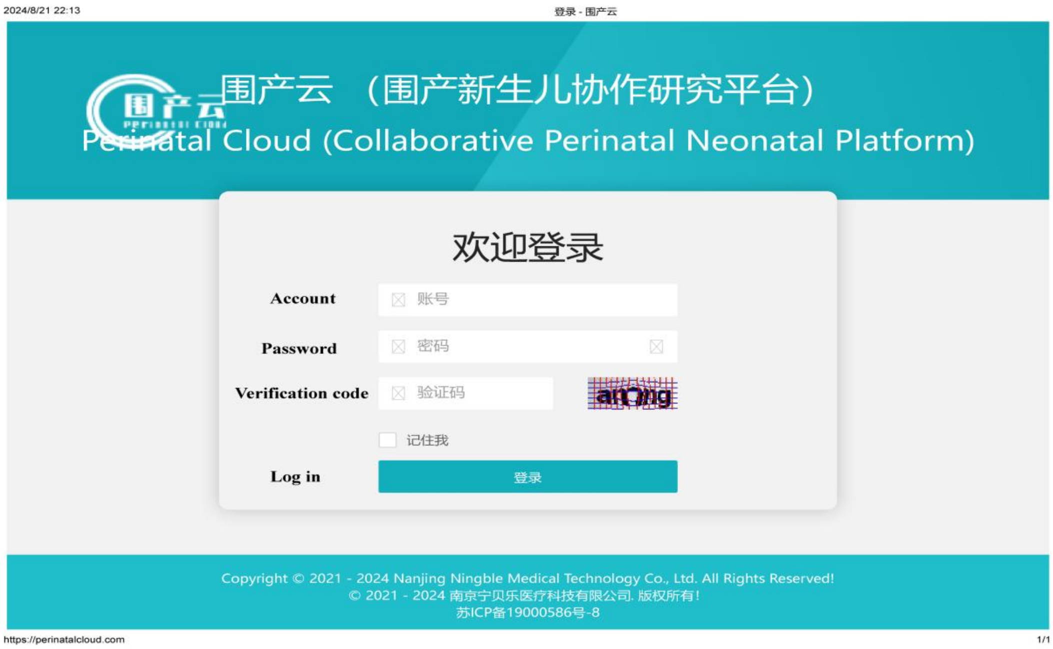

STEP 2 Add new case.

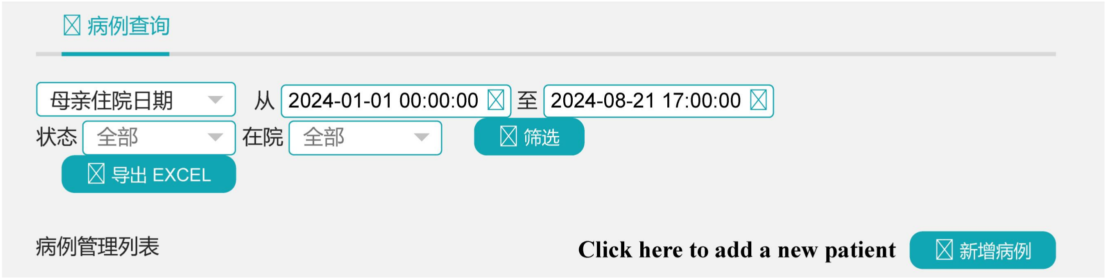

STEP 3 Fill in the demographic data and clinical information of the participates.

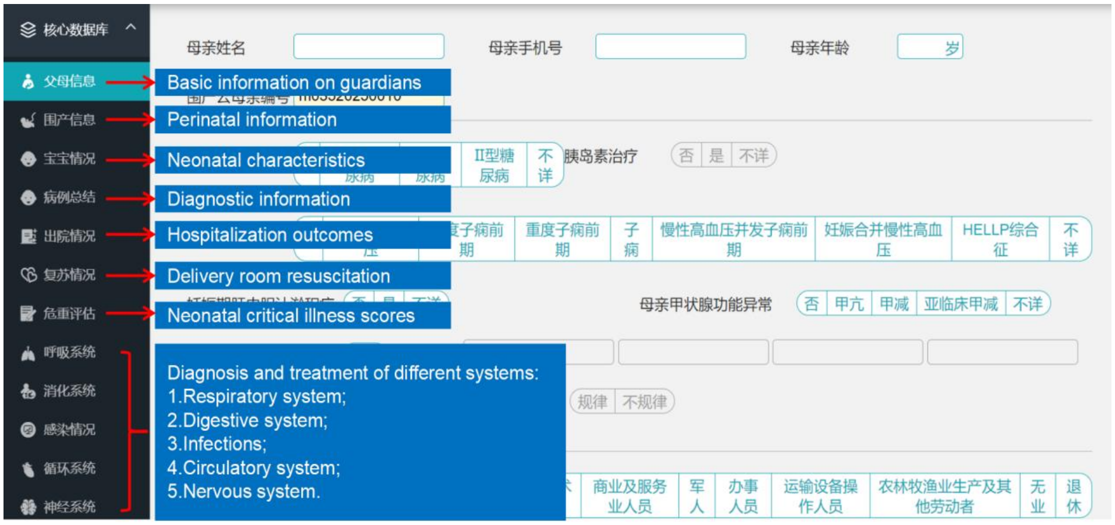

STEP 4 Date quality management.

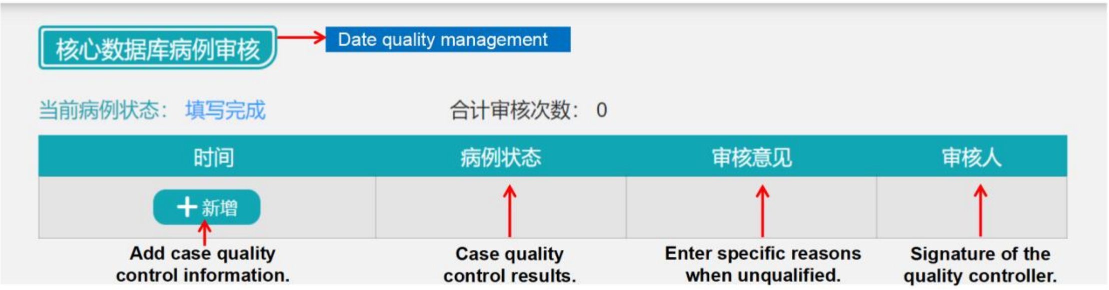

Supplement: Supplementary file 1 [file Datasheet1.pdf]
